# Molecular Cloning of Dynein Heavy Chain and the Effect of Dynein Inhibition on the Testicular Function of *Portunus trituberculatus*

**DOI:** 10.3390/ani11123582

**Published:** 2021-12-17

**Authors:** Qiumeng Xiang, Chaoguang Wei, Xinming Gao, Yiner Chen, Daojun Tang, Junquan Zhu, Congcong Hou

**Affiliations:** Key Laboratory of Applied Marine Biotechnology of Ministry of Education, School of Marine Sciences, Ningbo University, Ningbo 315832, China; 1911091064@nbu.edu.cn (Q.-M.X.); thomas.wei@outlook.com (C.-G.W.); nbugxm4851@163.com (X.-M.G.); chenyiner@nbu.edu.cn (Y.-E.C.); tangdaojun@nbu.edu.cn (D.-J.T.); zhujunquan@nbu.edu.cn (J.-Q.Z.)

**Keywords:** dynein, dynein heavy chain, testis, apoptosis, cell dysfunction

## Abstract

**Simple Summary:**

*Portunus trituberculatus* is a very important marine economic species. The study of its reproductive biology can provide a theoretical basis for its breeding. Dynein is a member of the motor protein family. It plays an important role in various life activities, such as cell division and intracellular material transport. In order to study the role of dynein in the testis of *Portunus trituberculatus*, we cloned the heavy chain of dynein and used the dynein inhibitor sodium orthovanadate to make the dynein lose its function. By detecting the localization of dynein, as well as the detection of various apoptosis indexes, antioxidant stress indexes and immune indexes, this study proved that dynein is essential in testis.

**Abstract:**

Dynein is a motor protein with multiple transport functions. However, dynein’s role in crustacean testis is still unknown. We cloned the full-length cDNA of cytoplasmic dynein heavy chain (*Pt-dhc*) gene and its structure was analyzed. Its expression level was highest in testis. We injected the dynein inhibitor sodium orthovanadate (SOV) into the crab. The distribution of *Portunus trituberculatus* dynein heavy chain (*Pt*-DHC) in mature sperm was detected by immunofluorescence. The apoptosis of spermatids was detected using a TUNEL kit; gene expression in testis was detected by fluorescence quantitative PCR (qPCR). The expression of immune-related factors in the testis were detected by an enzyme activity kit. The results showed that the distribution of *Pt*-DHC was abnormal after SOV injection, indicating that the function of dynein was successfully inhibited. Apoptosis-related genes *p53* and *caspase-3*, and antioxidant stress genes *HSP70* and *NOS* were significantly decreased, and anti-apoptosis gene *bcl*-*2* was significantly increased. The activities of superoxide dismutase (SOD) and alkaline phosphatase (AKP) were significantly decreased. The results showed that there was no apoptosis in testicular cells after dynein function was inhibited, but the cell function was disordered. This study laid a theoretical foundation for the further study of apoptosis in testis and the function of dynein in testis and breeding of *P. trituberculatus*.

## 1. Introduction

*Portunus trituberculatus* is an important aquatic economic animal in China. The basic reproductive biology of *P. trituberculatus* is a hot research field. The testis is the site of sperm production, and the production of crustacean sperm is often accompanied by nuclear deformation and material transport [1,2]. In these changes, motor protein plays an important role. Motor proteins are divided into microtubule-dependent kinesin and dynein, microfilament-dependent myosin. Motor proteins take the cytoskeleton as the motion orbit in the cell, and through the energy generated by the hydrolysis of ATP, and efficiently and accurately transports the carried goods to specific subcellular locations to perform physiological functions. [3]. The molecular mechanisms of many kinesins (such as KIFC1, KIF3A/3B, KIFC3, KIF-1, and KLC3) [4,5,6,7,8] and myosins (such as Myosin I, Myosin II, Myosin V/Va, Myosin VI, Myosin VII, and Myosin X) [9] in spermatogenesis have been gradually elucidated. However, the role of dynein in the male reproductive system has rarely been reported.

Unlike kinesin and myosin, which act as monomers or dimers, dynein is a giant protein complex consisting of heavy chains, intermediate chains, intermediate light chains, and light chains. Among them, the dynein heavy chain (DHC) exists in the form of dimer and has the activity of motor protein [10,11]. The dynein intermediate chain (DIC) is considered to be connected to the adaptor protein of anchoring cargo [12]. The dynein family includes cytoplasmic and axonemal dynein [13]. Axonemal dynein is a protein that exists in cilia or flagella and forms the lateral arm of the outer tubulin. Cytoplasmic dynein exists widely in cells, and its function is indispensable. In cells, cytoplasmic dynein transports mRNAs, endosomes, peroxisomes, autophagosomes, liposomes, mitochondria, and even viruses [14,15]. Studies have shown that the deletion of cytoplasmic dynein subunits or its regulatory factors can lead to neurodevelopmental abnormalities or neurodegenerative diseases [16]. In mice, deletion of the cytoplasmic dynein submit genes also leads to the death of embryos [17].

There are few reports on the role of cytoplasmic dynein in testis. Previous studies have proved that cytoplasmic dynein is highly expressed in the perinuclear nucleus during spermatogenesis in rats, and can be attached to the nuclear membrane and slide along the microtubule structure in the manchette to facilitate the formation of the fusarium nucleus [18]. During the development of *Drosophila melanogaster* spermatocytes, the intermediate chain gene *Dic61B* and light chain gene *tctex-1* play an important role in the connection between spermatocyte nucleus and flagellum base, and their deletion will lead to the loss of sperm motility and cause infertility [19,20].

The purpose of this study was to investigate the function of dynein in the testis of *P. trituberculatus*. Dynein is an extraordinarily sophisticated complex; therefore, we chose to investigate the function of dynein heavy chain (DHC, which is responsible for hydrolyzing ATP and binding to microtubules) in this study. SOV is a dynein inhibitor, which can inhibit dynein function by inhibiting the combination of ATP of DHC motor domain, thus preventing dynein from transporting substances along the microtubule [21,22]. We intend to inject dynein inhibitor SOV into *P. trituberculatus* to study the function of dynein in testis. We cloned and characterized the dynein heavy chain of *P. trituberculatus* (*Pt-dhc*), and studied its expression pattern. Immunofluorescence was used to detect the distribution of dynein in sperm cells after dynein function inhibited; qPCR was used to detect the expression of apoptosis-related genes, antioxidant stress genes; enzyme activity kit was used to detect immune correlation expression in testis and its impact on the reproductive system. This research was the first to study the effect of inhibition of dynein on testicular function of *P. trituberculatus*, which laid a theoretical foundation for the study of dynein’s function in testis and crab breeding.

## 2. Materials and Methods

### 2.1. Animals and Tissues

The *P. trituberculatus* in this experiment were selected from the QiXin farm of Ningbo Zhejiang, China. Healthy male *P. trituberculatus* subjects with the same age and similar growth conditions were selected for the experiment. The heart, muscle, hepatopancreas, gill, vas deferens and testis of *P. trituberculatus* were dissected in the laboratory, immediately immersed in liquid nitrogen, and then stored at −80 °C for subsequent experiments. 

### 2.2. SOV Injection and Sample Collection

Forty healthy male *P. trituberculatus* were randomly selected, with six in each group. The concentrations of 0, 0.5, 2, 4 and 8 μg/g SOV (Solarbio, Beijing, China) were injected, respectively, and cultured in an 80 L storage box for 48 h. The heart, testis, hepatopancreas, muscle and gill tissues of *P. trituberculatus* at various concentrations were aseptically dissected. Part of the testis tissue was fixed with 4% paraformaldehyde in 0.1 M PBS (pH 7.4), embedded in O.C.T. compound (SAKURA, Torrance, CA, USA), formed in frozen section-embedded blocks and stored at −80 °C.

### 2.3. Total RNA Extraction and Reverse Transcription of cDNA

Total RNA was extracted from heart, muscle, hepatopancreas, gill, vas deferens and testis by RNA solv Reagent (OMEGA, Norcross, GA, USA). The cDNA used for intermediate fragment cloning was obtained by the PrimeScript^®^ RT reagent Kit (Takara, Dalian, China) and the cDNA used for rapid amplification of cDNA end (RACE) was obtained by SMARTer RACE 5′/3′ Kit (Takara) and 3′-Full RACE Core Set with PrimeScript™ RTase (Takara). HiFiScript gDNA Removal cDNA Synthesis Kit (Cwbio, Beijing, China) was used to synthesize cDNA for qPCR. All the cDNA was stored at −80 °C.

### 2.4. Full-Length cDNA Cloning of Pt-dhc

First, we downloaded the dynein heavy chain 1(*dhc*) cDNA sequences of *Homo sapiens* (NM_001376.3), *Mus musculus* (NM_030238.1), *Danio rerio* (DQ323903.1), *Xenopus tropicalis* (XM_012961507.1) and *Drosophila melanogaster* (BC154077.1) from the National Center for Biotechnology Information (http://www.ncbi.nlm.nih.gov/, accessed on 1 November 2016, NCBI). Multiple sequence alignment was completed using Vector NTI 11.5 (Invitrogen, Waltham, MA, USA), conservative region base sequences were selected, and degenerate primers were designed with Primer Premier 5.0 software (Premier Biosoft International, Palo Alto, CA, USA) for intermediate fragment cloning (the primer sequences used in this study are shown in Table 1). PCR products were isolated from the 1% agarose gel (with 0.1% nucleic acid dye added), the correct nucleic acid bands were cut immediately and the target DNA fragment was recovered using an Agarose Gel DNA Extraction Kit (Takara). The DNA fragment was connected to the pMD-19T vector (Takara) and then transfected into DH5α Competent Cells (Takara). The correctly connected cells were identified by PCR using M13F/R primers. The cells identified correctly were sent to the Beijing Genomics Institute (Shanghai, China) for sequencing. After obtaining the intermediate fragment sequence, the specific primers (Table 1) for RACE were designed using Primer Premier 5.0 software. The PCR products were recovered by the same operation as above to obtain 5′ cDNA fragment sequences and 3′ cDNA fragment sequences. The 5′ cDNA fragment sequences and 3′ cDNA fragment sequences were spliced with the intermediate fragment sequence to obtain the full length of *Pt-dhc*.

### 2.5. Sequence Analysis and Structure Prediction

The primary structure of *Pt*-DHC protein was predicted using online tools (http://www.bio-soft.net/sms/, accessed on 18 April 2021). The molecular weight and isoelectric point of *Pt*-DHC protein were predicted by ExPASy ProtParam tool (http://web.expasy.org/protparam/, accessed on 18 April 2021). DHC protein sequences of *Homo sapiens* (NP_001367.2), *Mus musculus* (NP_084514.2), *Danio rerio* (NP_001036210.1) and *Drosophila melanogaster* (NP_001261430.1) were downloaded from NCBI, and multiple sequence alignment was performed using Vector NTI 11.5 (Invitrogen). The phylogenetic tree of DHC protein amino acid sequences of different species was constructed using mega 5.1 software; the GenBank accession numbers of DHC homologues were *Penaeus vannamei* (XP_027209046.1), *Athalia rosae* (XP_012264199.1), *Apis dorsata* (XP_006622931.1), *Hyalella Azteca* (XP_018024055.1), *Gallus gallus* (XP_015143281.1), *Cryptotermes secundus* (XP_023724922.1), *Pogona vitticeps* (XP_020649242.1), *Lonchura striata domestica* (XP_021405311.1), *Sander lucioperca* (XP_031166063.2) and *Larimichthys crocea* (XP_027130998.1). 

### 2.6. Quantitative mRNA Analysis 

The specific primers of *Pt-dhc*, *p53* (MH155953.1), *caspase-3* (KY406168.1), *NOS* (KU306112.1), *HSP70* (FJ830635.1) and *GAPDH* (EU919707.1) genes were designed using the Primer Premier 5.0 software tool (Table 1). The mRNA expressions levels of those genes were detected by qPCR using 2 × RealStar Green Fast Mixture kits (Genstar, Beijing, China). The sample size was 5 and the number of experimental replicates was three. The reaction system contained 1 μL of cDNA template, 0.5 μL of forward and reverse primers, 10 μL of 2 × RealStar Green Fast Mixture, 0.4 μL of ROX Reference Dye/ROX Reference Dye II***, and double-distilled water to 20 μL. The qPCR program was completed with pre-denaturation at 95 °C for 2 min and 40 cycles (denaturation at 95 °C for 15 s, annealing at 60 °C for 30 s, extension at 72 °C for 30 s). With the housekeeping gene, *GAPDH,* as the internal reference gene, the relative expression of *Pt-dhc* mRNA was compared based on the ΔΔCt method, and the data were analyzed by one-way analysis of variance using the SPSS v20.0 software.

### 2.7. Immunofluorescence and Apoptosis Detection

The frozen slicer was used to make the embedded tissue into 5 μm-thick slices, and the polylysine-treated glass slides were used to adhere the frozen slices and stored at −80 °C. The frozen slices were taken out to dry at room temperature for 10 min; then, the dried slices were placed in 0.3% PBST (0.3% Triton X-100 in 0.1 M PBS) and allowed to stand at room temperature for 10 min. 

For apoptosis detection, the slides were washed twice with 0.1M PBS, stained with TUNEL staining solution (the staining solution was configured according to the instructions using the TUNEL kit (Beyotime, Dalian, China)), washed three times with 0.1M PBS, added to the antifade mounting medium (Beyotime), and sealed with nail polish. The reaction was observed and photographed using a laser confocal microscope (ZEISS LSM880).

For immunofluorescence, the slides were blocked in antibody blocking buffer (1% BSA in 0.1% PBST) for 1 h; next, slices were incubated with rabbit anti-*Pt*-DHC antibody (1:50 dilution; Abcam, Cambridge, UK) in antibody blocking buffer overnight at 4 °C, and then washed three times in 0.1% PBST for 10 min each time. The slices were then incubated with Alexa Fluor 488 goat anti-rabbit IgG (1:500 dilution; Beyotime) in antibody blocking buffer for 30 min and washed three times in 0.1 M PBS for 10 min each time. A DAPI staining solution (Beyotime) was added to stain the nucleus for 5 min and Antifade Mounting Medium was used to seal and observe (the same as the method used for apoptosis detection).

### 2.8. Enzyme Activity Test

The testes of *P. trituberculatus* were homogenized with normal saline on ice and centrifuged at 3000 rpm at 4 °C for 10 min. The supernatant was extracted and the protein concentration was measured using a BCA kit (Njjcbio, Nanjing, China). The activities of SOD, AKP and ACP in the supernatant were analyzed according to the instructions of the enzyme activity Kit (Njjcbio).

## 3. Results

### 3.1. Full Length of Pt-dhc cDNA Sequence and Protein Structure

We obtained the full-length cDNA sequence of *Pt-dhc* (GenBank: MF476875.1, see Figure S1 of Supplementary File for more details) through RACE technology. The *Pt-dhc* cDNA has a total length of 14,219 bp, encoding 4639 amino acids, an 87 bp untranslated region at the 5′ end and a 212 bp untranslated region at the 3′ end. The calculate molecular weight of *Pt*-DHC was 529.3 kDa and the theoretical pI was 6.0. 

**Figure 1:**
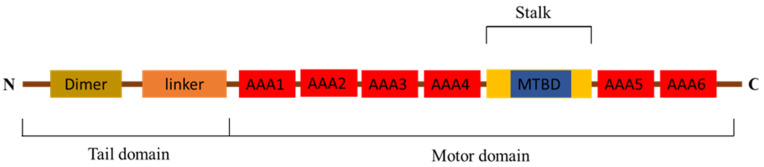
The protein structure domain of *Pt*-DHC. *Pt*-DHC divided into tail domain and motor domain, consisting of 6 AAA structures, MTBD, dimer and linker.

The protein structure domain of *Pt*-DHC shows that *Pt*-DHC can be divided into tail domain and motor domain (Figure 1). The tail domain contains a dimer and linker; the motor domain includes six AAA structures and microtubule binding domain (MTBD). 

### 3.2. Sequence Alignment and Phylogenetic Analysis

**Figure 2:**
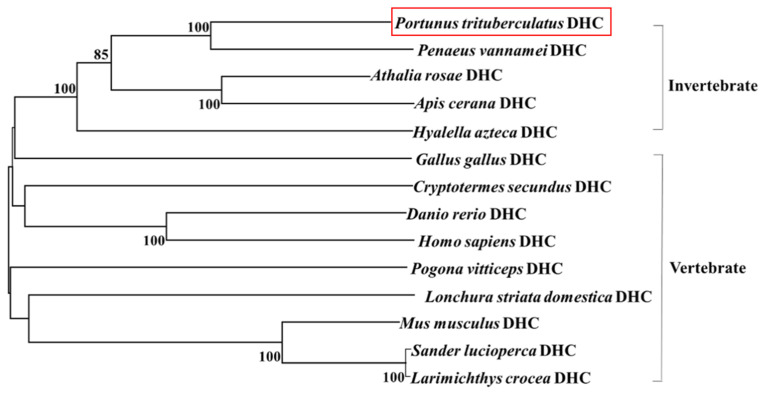
Phylogenetic tree based on DHC protein sequences. The phylogenetic tree was generated based on the alignment of proteins sequences using MEGA 5.0 software using neighbor-joining method.

The protein sequence alignment results show that *Pt*-DHC had 90.15%, 80.56%, 76.30% and 76.20% identity with its homologs in *Penaeus vannamei*, *Hyalella azteca*, *Athalia rosae* and *Apis cerana*, in invertebrates, respectively, (Figure S2 of Supplementary File). We analyzed the phylogenetic relationship of DHC using the neighbor joining method using Mega V5.0 software (Figure 2) and *Pt*-DHC clustered with the invertebrate. In comparison with the DHC of various species, *Pt*-DHC has the closest evolutionary relationship with *Penaeus vannamei* DHC (Figure 2).

### 3.3. The Expression of Pt-dhc in Different Tissues of P. trituberculatus

**Figure 3:**
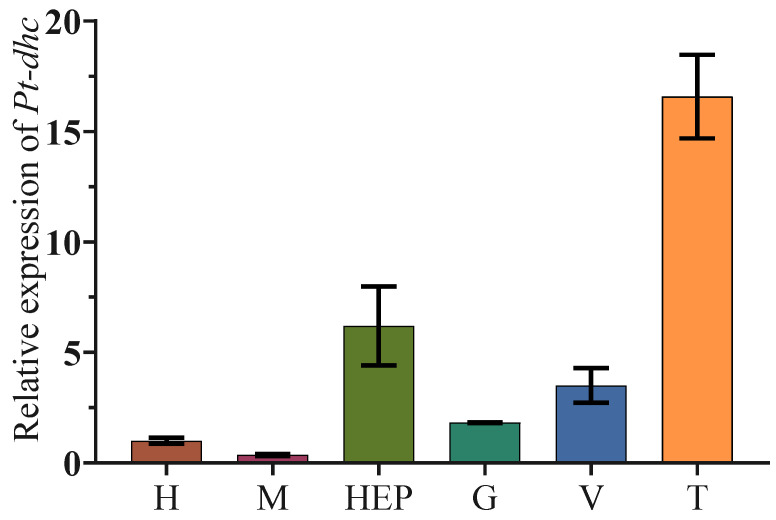
Expression level of *Pt-dhc* in different tissues. The *Pt-dhc* is widely expressed in different tissues in *P. trituberculatus*, with the highest expression in the testis. H: heart; M: muscle; Hep: hepatopancreas; G: gill; V: vas deferens; T: testis.

We also analyzed the expression of *Pt-dhc* mRNA in heart (H), muscle (M), hepatopancreas (Hep), gill (G), vas deferens (V) and testis (T) of *P. trituberculatus*. The qPCR results of *Pt-dhc* show that *Pt-dhc* mRNA is widely expressed in all organizations examined and has the highest expression level in the testis (Figure 3). 

### 3.4. The Expression and Distribution of Pt-DHC in the Testis of P. trituberculatus Changed after Injection of SOV

**Figure 4:**
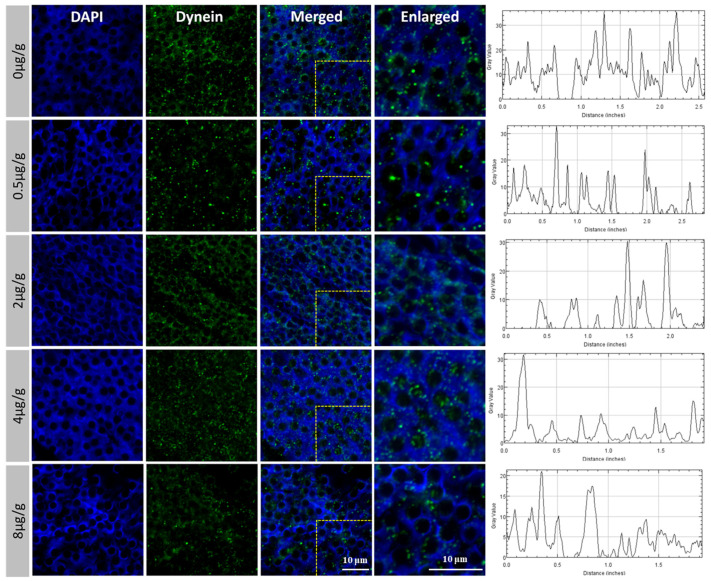
*Pt*-DHC distribution of mature sperm in the testis of *P. trituberculatus* treated with different concentrations of SOV. In the control group and 0.5 μg/g group, dynein is mainly distributed at the front end of the acrosome. Additionally, in the 2.0, 4.0 and 8.0 μg/g groups, dynein is dispersed in cytoplasmic regions. Green fluorescence is dynein (*Pt*-DHC) and blue fluorescence is the nucleus. Bar = 10 μm.

In order to further explore how *Pt*-DHC influences the development of the testis and spermatogenesis, we inhibited the activity of *Pt*-DHC by injecting different concentrations of SOV, conducting immunofluorescence on the *Pt*-DHC of *P. trituberculatus* testis. The results show that, in the control group and 0.5 μg/g group, *Pt*-DHC was mainly distributed in the front end of the acrosome of mature sperm. As the concentration of the inhibitor increased, the signal of *Pt*-DHC was obviously weakened and the distribution of *Pt*-DHC became more dispersed (Figure 4).

### 3.5. Detection of Testicular Cell Apoptosis in the Testis of P. trituberculatus after SOV Injection

**Figure 5:**
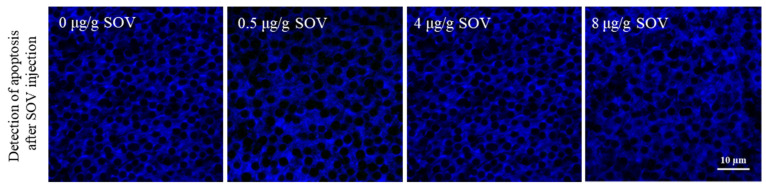
Apoptosis detection. No obvious signal (FITC, green fluorescence) was detected after injection of different concentrations of SOV in testis.

We used the TUNEL kit to explore whether apoptosis occurred in testis after SOV injection. The results showed that no obvious apoptosis signal (FITC, green fluorescence) was detected 48 h after the injection in 0, 0.5, 4 and 8 μg/g SOV concentration groups. The results showed that there was no apoptosis in the testis after injection of SOV (Figure 5).

### 3.6. Dynein Inhibited by SOV Significantly Reduced the mRNA Expression Level of Genes Related to Apoptosis in Testis of P. trituberculatus

**Figure 6:**
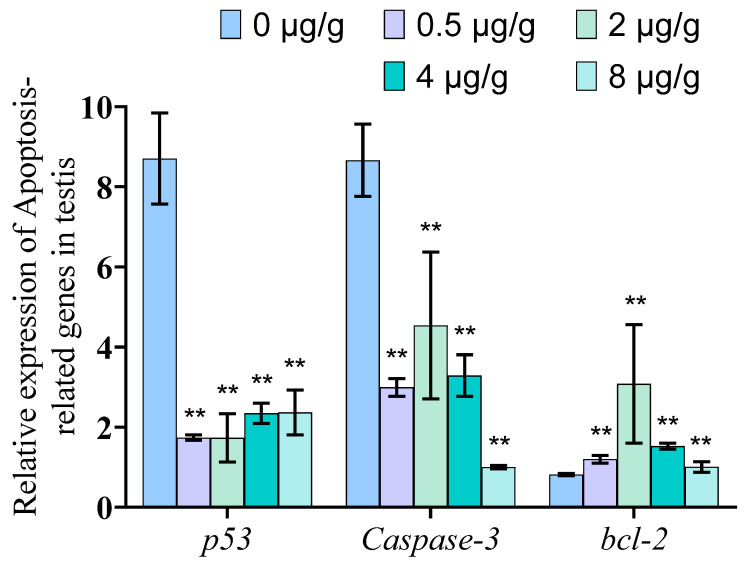
Apoptosis-related genes expression. The expression of *p53* and *Caspase-3* mRNA in the testis decreases significantly and *bcl-2* mRNA increases significantly after SOV injection (** indicates significant compared with control group Significance. **: *p* < 0.01).

To investigate whether the suppression of dynein via SOV injection would induce changes in the level of apoptosis of the testis, we measured the expression levels of apoptosis-related genes *p53*, *Caspase-3* and *bcl-2*. The qPCR results showed that the expression levels of *p53* and *Caspase-3* genes decreased significantly (*p* < 0.01), and the expression level of the *blc-2* gene increased significantly (*p* < 0.01) in *P. trituberculatus* testis after SOV injection (Figure 6).

### 3.7. Dynein Inhibited by SOV Changes the Expression of Anti-Oxidative Stress Genes

**Figure 7:**
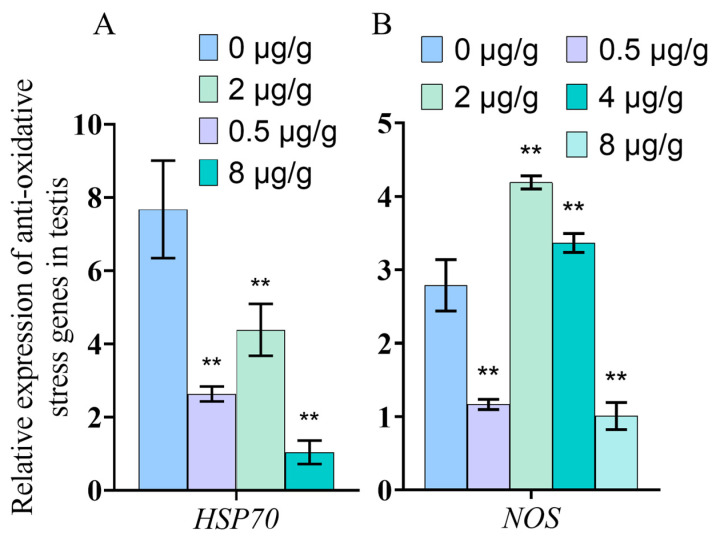
Anti-oxidative stress gene expression. (**A**) The expression of the *HSP70* gene in the testis decreased significantly. (**B**) The expression of the *NOS* gene in the testis decreased significantly in the 0.5 μg/g and 8 μg/g SOV groups, but increased significantly in the 2 μg/g and 4 μg/g groups. (** indicates significant compared with control group; **: *p* < 0.01).

The qPCR results show that the expression of anti-oxidative stress genes *HSP70* and *NOS* changed in tissues of *P. trituberculatus* after SOV injection (Figure 7). The results showed that the expression level of *HSP70* decreased significantly (*p* < 0.01) in the testis tissue. The expression level of *NOS* gene decreased significantly at 0.5 μg/g (*p* < 0.01) and 8 μg/g (*p* < 0.01), but increased significantly at 2 μg/g (*p* < 0.01) and 4 μg/g (*p* < 0.01) (Figure 7). 

### 3.8. Dynein Inhibited by SOV Induced the Changes in SOD and AKP Enzyme Activities of P. trituberculatus Tissues

**Figure 8:**
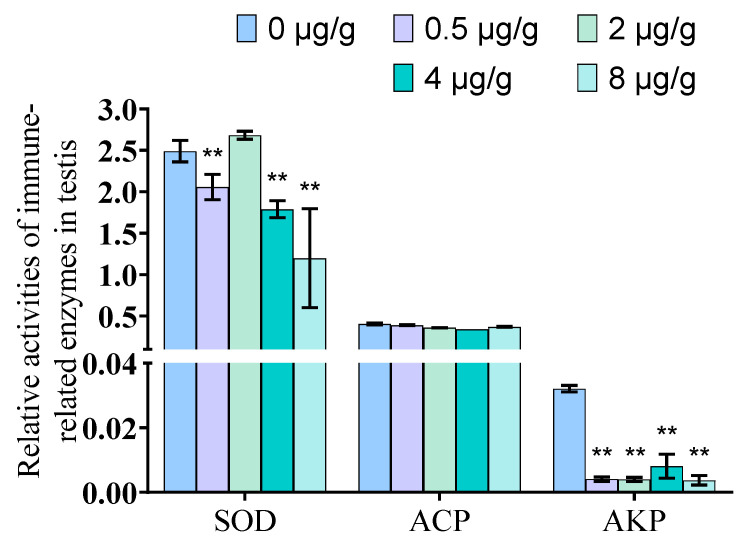
Enzyme activity detection of immunity-related factors. The enzyme activities of SOD and AKP in the testis decrease significantly, and ACP was no significant changed (** indicates significant compared with control group Significance. **: *p* < 0.01).

The results show that the SOD and AKP enzyme activities decreased significantly after SOV injection compared with the control group (*p* < 0.01), while the ACP enzyme activity of the experimental group did not change significantly compared with the control group (Figure 8).

## 4. Discussion

### 4.1. Protein Structure and mRNA Expression Characteristics of Pt-dhc 

Cytoplasmic dynein is a multi-subunit motor complex with huge molecular weight, which performs a variety of cargo transport functions in cells. The DHC dimer is the core of cytoplasmic dynein. This is a multifunctional that is not only responsible for connecting the other components of other dynein complexes, such as intermediate chain (DIC) and regulatory proteins such as LIS1, but also binds to microtubules via the microtubule-binding domain (MTBD) and provides power for the negative movement of dynein complexes along microtubules by hydrolyzing ATP by six AAA ATPase domains. The other components of the dynein complex, such as the DIC and the light chain (DLC), are completed by the assembly of the dynein complex or combined with “goods” such as organelles and vesicles [23]. Moreover, DHC can change the function of dynein complex by binding with different components [24,25]. Therefore, studying DHC can help further elucidate the function of dynein.

In this study, we cloned the full length of *Pt-dhc* cDNA. Through a comparison with other invertebrates and vertebrate DHC amino acid sequences (Figure S2), we found that DHC is very conservative in its evolution. The structure of *Pt*-DHC is very similar to that of DHC in other species. *Pt*-DHC divides into a tail domain and motor domain—the tail domain contains dimer and linker, and the motion domain contains six AAA structures and MTBD, consistent with the structures found in human and mouse DHC [24]. Studies have shown that DHC has a conserved function, which operates in the same way in a diverse range of organisms, from algae to humans [25]. Therefore, we speculate that *Pt*-DHC has similar functions to the DHC of other species and plays an important role in testis function. 

In this study, we found that *Pt-dhc* mRNA was widely expressed in the studied tissues, with the highest expression in testis. DHC is widely expressed in mammalian tissues, with the largest expression in the brain and testis [26]. In previous studies, dynein plays an important role in the brain and is responsible for the survival of neurons in mammals. Many neuronal defect diseases are related to the mutation of dynein, which proves that dynein plays an important role in brain development and the maintenance normal functioning [27,28]. This explains why DHC is highly expressed in the brain. In mammalian testis, spermatogenesis is often accompanied by cell deformation, chromosome separation, organelle and protein transport, etc. As a member of the molecular motor, dynein provides power for these life activities [26]. The high expression of *Pt-dhc* mRNA in the testis of *P. trituberculatus* suggests that dynein may be related to the development of *P. trituberculatus* testis or spermatogenesis. The specific role of dynein in the spermatogenesis of *P. trituberculatus* requires further exploration.

### 4.2. The Function of Dynein Was Inhibited after SOV Injection

In this study, we observed that in the normal mature sperm of *P. trituberculatus*, the *Pt*-DHC signal was mainly concentrated at one end of the mature sperm (Figure 4). Previous studies have proved that this is the location of the acrosome of the mature sperm of *P. trituberculatus* [29,30,31]. In our previous studies, it has been proven that dynein is mainly located on the acrosome in the mature sperm of *P. trituberculatus*, which may be related to the formation of acrosome and the transport of Golgi vesicles [32]. In this study, *Pt*-DHC is mainly distributed in the front end of the acrosome of material sperm in the testis section of the control group, which is consistent with previous studies (DHC is an indispensable component of dynein; therefore, its fluorescence signal can be regarded as the fluorescence signal of dynein).

SOV can inhibit cytoplasmic dynein ATPase activity. SOV makes dynein lose its movement force, inhibiting the ability of dynein to transport material [33,34]. In order to verify whether SOV can inhibit the function of dynein in *P. trituberculatus*, we observed the localization of dynein in mature sperm 48 h after the injection of different concentrations of SOV by immunofluorescence; while after SOV injection, the intracellular localization of *Pt*-DHC changed and was no longer focused on the acrosome, which means that dynein’s transport function is inhibited and can no longer be positioned correctly. The results showed that injection of SOV could effectively inhibit the function of dynein in *P. trituberculatus*.

### 4.3. Dynein May Indirectly Participate in Cell Apoptosis in the Testis 

Apoptosis is the main process by which programmed cell death occurs in multicellular organisms to remove damaged and harmful cells, such as cancerous cells [35]. *P53* is an important tumor suppressor gene. When cellular stress and damage occurs, *p53* will be up regulated to induce apoptosis and other effector processes. Therefore, the expression level of *p53* is often used as an apoptosis index to reflect the degree of apoptosis [36]. The *Caspase-3*-mediated signaling pathway is necessary for most apoptosis processes, and Caspase-3 is the executor of apoptosis [37]. Therefore, the expression of *Caspase-3* also can directly reflect the degree of apoptosis [37]. *Bcl-2* is an important anti-apoptosis gene, which acts on the upstream of Caspase-3. The Bcl-2 protein is the substrate of the Caspase-3 protein. These proteins regulate each other with *Caspase-3*. When apoptosis occurs, its expression is inhibited [38,39]. In order to understand the role of dynein in apoptosis, we studied the degree of apoptosis in testis after dynein function was inhibited. Theoretically, the inhibition of dynein function will lead to cell damage. Therefore, apoptosis should occur in testis, and *p53* and *Caspase-3* genes will be significantly up regulated; the expression of *bcl-2* will be significantly down regulated. However, the results showed that, following SOV injection, no obvious apoptosis signal (green) was detected in the testis (Figure 5), indicating that there was no apoptosis in the testis. Furthermore, the expression of *bcl-2* significantly increased, and the expression levels of *p53* and *Caspase-3* significantly decreased. This outcome was the opposite of our hypothesis (Figure 6). The results showed that when dynein function was inhibited, apoptosis did not occur. Dynein plays an irreplaceable transport role in cells [14,15]. Its function in cells is indispensable. If dynein is lacking, neurological diseases and even embryo death will occur [16,17]. The following question remains: why does apoptosis not occur in testis when dynein function is impaired? Previous studies have proven that P53 is transported by dynein [40,41,42] (P53 is connected to dynein light chain LC8 by HSP90, and then transported to the nucleus by dynein to regulate the expression of related genes [41,42]), and Caspase-3 is also transported by dynein [43]. However, at present, no evidence has been found to prove that Bcl-2 is transported by dynein. To explain why *Caspase-3* and *p53* were down regulated and *bcl-2* was up regulated after dynein inhibition, we suggest that Caspase-3 and P53 were not able to be transported to the correct location. As Caspase-3 is the main effector of apoptosis [37] and *p53* is an important apoptosis-regulated gene, when dynein cannot function normally, the process of apoptosis cannot proceed even if the cells are damaged. While Bcl-2 is not transported by dynein, and since apoptosis did not occur, the expression of *bcl-2* rises in response to the tendency of apoptosis to decrease. In addition to transporting normal substances, dynein is also related to the clearance of faulty proteins [11,23]. If dynein fails, a large accumulation of faulty proteins occurs, affecting the function of cells. This is the other possible reason that there was no apoptosis in testicular cells. In conclusion, the fact that apoptosis did not occur after dynein function was inhibited means that dynein may play an important role in the apoptosis of testis. At present, there are few studies on the relationship between dynein and apoptosis. Some researchers found that dynein light chain 1 (LC8) is related to apoptosis that LC8 binds to BIM of Bcl-2 family in the regulation of the mitochondrial pathway of apoptosis. However, the function of LC8 affecting apoptosis seems to be independent of the dynein complex [44,45,46]. In addition, other studies have found that LC8 and DHC are located together, localized in the nuclear membrane inside apoptotic germ cells in *C. elegans*. LC8 and DHC jointly regulate germ cell apoptosis, and germ cell apoptosis is inhibited when LC8 is absent [47]. The absence of apoptosis in testicular cells after dynein inhibition does not mean that testis damage is not present, but suggests that apoptosis cannot proceed normally after cell injury. Therefore, we hypothesize that dynein may indirectly participate in cell apoptosis in the testis, and that apoptosis is very important for spermatogenesis and the removal of faulty sperm to ensure reproductive ability in males. The specific mechanism of dynein in apoptosis and the role of dynein in other tissues requires further research.

### 4.4. Inhibition of Dynein Function May Lead to Cell Dysfunction in Testis

HSP70 is an important member of the HSP family. It can reduce cell damage and protect cells by increasing its expression in response to external stimuli [48]. NOS is an important enzyme for the synthesis of NO, and NO is involved in nonspecific immunity in addition to being a neurotransmitter. Therefore, *NOS* is also an important gene that protects cell function [49,50]. To explore whether cell function is impaired when dynein function is inhibited, we analyzed *HSP70* and *NOS* mRNA expression in testis after dynein function inhibited by qPCR. The mRNA expression of *HSP70* and *NOS* can reflect the antioxidant stress ability of organisms when stimulated. The results show that *HSP70* expression was decreased after SOV injection (Figure 7A). However, interestingly, with the increase in SOV concentration, the mRNA expression of *NOS* decreased first, then increased, and then decreased compared with the control group (Figure 7B). We speculate that when the transport capacity of dynein is slightly reduced, the ability of cells to cope with external stimuli decreased, resulting in a decrease in *NOS* expression. With the increase in the inhibition degree of dynein, the cells were damaged. Therefore, the expression of *NOS* is up regulated to protect the cells. However, when the function of dynein is almost completely inhibited, cell function was disordered, resulting in a strong inhibition of *HSP70* and *NOS* expression regulation. 

As an invertebrate, *P. trituberculatus* only has innate immunity; unlike vertebrates, it does not have specific immunity. In order to study whether the testicular immune system is damaged after the function of dynein is inhibited, we also detected the activities of immune-related enzymes ACP, AKP, and SOD in the testis. ACP and AKP are enzymes with immune function and an important detoxification system in animals. They can be used together as an index enzyme to detect the immune function of crustaceans [51,52]. An antioxidant system is an important system used by organisms to eliminate excess reactive oxygen species (ROS) and avoid oxidative damage. SOD is an important antioxidant enzyme. Its activity can reflect antioxidant capacity [53]. The results showed that the activity levels of SOD and AKP in the testis were decreased. The levels of ACP did not change significantly (Figure 8). The results showed that the testicular immune system was damaged after dynein failure. At present, there is no relevant report on the relationship between dynein and ACP, AKP and SOD. This study shows that, when the function of dynein is inhibited, immune function will decline and dynein has a certain relationship with SOD and AKP, which is preliminary proven by the fact that dynein plays an important role in testis health. However, the specific role of dynein in this process requires further research.

## 5. Conclusions

In this study, we cloned *Pt-dhc* from *Portunus trituberculatus*. Our study proved that *Pt*-DHC is very conservative in evolution, which is widely expressed in various tissues of *P. trituberculatus*. The highest expression of dynein occurs in testis, suggesting that it has an important function in spermatogenesis. By locating the cell subcellular localization of *Pt*-DHC, we proved that SOV successfully inhibited the function of dynein. We found that when dynein functioning was inhibited, apoptosis did not occur in testis. Our study proved that dynein may indirectly participate in the apoptosis of *P. trituberculatus* testis. However, the specific mechanism involved in this process requires further research. By measuring the expression of antioxidant stress genes and immune-related enzyme activity in testis, we found that the inhibition of dynein function may lead to testicular cell dysfunction. 

## Figures and Tables

**Table 1:** The primer sequence used in *Pt-dhc* cDNA full-length cloning and qPCR.

Primer	Sequence (5′–3′)	Purpose
DHCF1	CGCAAGTTCCTGTCCGAYCCNCARRT	PCR
DHCF2	CCAACCTGCCCGACAAYYTNAARAA	PCR
DHCF3	CTTTGTCCAAGGAAGTGCGG	PCR
DHCF4	GCTGATCAAGGGCTACATGAAGRTNAAYATGYT	PCR
DHCF5	CGACACCGTGCTGATGGARCARCCNCC	PCR
DHCF6	GGAGCAGGGAGGAGGCAG	PCR
DHCF7	GACTCCGGCTTCCTGGAGMGNATGAAYAC	PCR
DHCF8	GCGGCAGACATTTCCCTCA	PCR
DHCF9	TGGCCGCCGAGCARAAYAMNCA	PCR
DHCR1	AGCACGCTGGATGGGGTA	PCR
DHCR2	CTCGACCTTCTCGCAGGTNCKYTCRTA	PCR
DHCR3	CGTCCTCCTCGAACACCTTRTARTANGG	PCR
DHCR4	CATAGCCAGGGAGCGGAAC	PCR
DHCR5	GTGGCGTACTTCAGGTCCTGNACYTCRAACA	PCR
DHCR6	CACGGGCACCTTGGGRTTDATYTCCA	PCR
DHCR7	TGAGGGAAATGTCTGCCGC	PCR
DHCR8	TCCTGGATGATGGCGTGRAACCANGC	PCR
DHCR9	AGGACGGCCACGCCNCKYTCRTA	PCR
5′DHCF1	TCACAGACTTCTCCCAGCGT	5′RACE
5′DHCF2	TTCACAGCAAGTGGCTCAGT	5′RACE
5′DHC5F3	AGAATGTAACGGTGTGGCTG	5′RACE
3′DHC3R1	CGGAGCAAAGGCATTGGCTACATA	3′RACE
3′DHC3R2	CTCCATCCCTTTCAATCTTGCCAGT	3′RACE
3′DHC3R3	AATAGGTAATGAGATGTCTGGGATGTCG	3′RACE
P53F	GGGTAACGCCATGAACGAGA	*P53* qPCR
P53R	GCTGCATCTCCGTGTGTTTC	*P53* qPCR
CAS3F	TCACAGATTGACAAAGAGCGG	*Caspase-3* qPCR
CAS3R	TCCTCAGGTCAGTAGTGGAAATG	*Caspase-3* qPCR
BCL2-F	AGCTTACAACTGGATGCGCT	*Bcl-2 qPCR*
BCL2-R	TCGAGAGTGATTTAGGCGGC	*Bcl-2 qPCR*
NOSF	GGAACCCTTCTGAGCAACGA	*NOS* qPCR
NOSR	CGTGTGTGGAGGTTGTCGTA	*NOS* qPCR
HSP70F	CTCAGATGGAGGCAAGCCAA	*HSP70* qPCR
HSP70R	CTTGACGGTAGTGCCCAAGT	*HSP70* qPCR
GAPDHF	TGAGGTGAAGGTAGAGGAT	Positive control of qPCR
GAPDHR	CCAGTGAAGTGAGCAGAG	Positive control of qPCR

## Data Availability

*Pt-dhc* nucleic acid sequence has been uploaded to NCBI (GenBank: MF476875.1).

## Supplementary Materials

The following are available online at https://www.mdpi.com/article/10.3390/ani11123582/s1, Figure S1: Nucleic acid sequence and translated protein sequence of *Pt*-*dhc*. Figure S2: Multiple sequence alignment of protein sequences of *Pt-dhc*.
