# Supplementary material for: Molecular Cloning of Dynein Heavy Chain and the Effect of Dynein Inhibition on the Testicular Function of Portunus trituberculatus
Source: Animals (Basel). 2021 Dec 17;11(12):3582. doi: 10.3390/ani11123582 (PMC8697902; doi:10.3390/ani11123582)
Supplement: Supplementary file 1 [file animals-11-03582-s001.zip › animals-1453464-supplementary.pdf]

1 gtgacaggcgagcaggggcggaacgaacaccttgcattctactcgaactactgcatctaaccactgcatacctgc

1 M D T G E E G D G G S G G L E T V V A V A

76 tgaggaggaacgATGGACACCGGTGAGGAGGGTGATGGAGGCAGTGGGGGGTTGGAGACCGTGGTGGCGGTGGCG

22 E V E A V A K H L Q R L S H V L L E E E D Q P S P

151 GAGGTGGAGGCGGTGGCCAAACACCTGCAGCGCCTCAGTCATGTGTTGCTGGAGGAGGAGGATCAGCCATCGCCT

47 E L V A A M K S P A V L E V T R K F V A D P Q T K

226 GAACTGGTGGCTGCCATGAAAAGCCCTGCAGTCCTGGAGGTGACACGCAAGTTTGTGGCTGATCCTCAGACCAAG

72 S I L I Q R S T T K E E T E E G A E V E E D K E V

301 TCAATCCTTATTCAGCGCAGCACCACTAAAGAGGAGACAGAAGAGGGAGCTGAAGTGGAGGAAGACAAGGAAGTG

97 V Y H I S S G V F F T S T K M S S L L C L K R G G

376 GTGTACCACATATCCAGTGGTGTTTTCTTCACCTCCACCAAGATGAGCAGCCTGCTGTGCCTCAAACGTGGTGGT

122 V V E A D K P V S A Q I R S W S L S E G S P Y E T

451 GTGGTGGAAGCAGACAAGCCTGTCAGTGCACAGATTCGCTCATGGAGTCTCAGTGAAGGTTCCCCTTATGAGACG

147 L H D Y V A N A F A P Y F K A Y V K E T G K I E R

526 CTGCATGACTATGTAGCCAATGCCTTTGCTCCGTACTTTAAGGCTTATGTGAAGGAGACTGGCAAGATTGAAAGG

172 D G D K M A P S V E K K I A E L E M G L L H L Q Q

601 GATGGAGATAAAATGGCACCATCAGTTGAGAAGAAGATTGCAGAGCTTGAGATGGGGCTGCTGCACTTGCAACAG

197 N I D I P D I S L P I H P T V A Q V I K M C A D E

676 AACATCGACATCCCAGACATCTCATTACCTATTCATCCAACTGTAGCACAGGTGATCAAGATGTGTGCAGACGAG

222 G T R P K V A D F G D R V E D S N L L N Q L Q N G

751 GGAACCCGGCCAAAGGTGGCTGACTTTGGGGATCGTGTTGAGGACTCCAACCTCCTCAACCAGCTGCAGAACGGT

247 V A R W I R E I Q K V T K L D R D P S S G T A L Q

826 GTGGCTCGATGGATCAGGGAGATACAGAAGGTGACCAAACTGGACCGAGATCCTTCAAGTGGCACAGCCCTGCAG

272 E I S F W L N L E R A L L K I Q D K R E S L E V A

901 GAGATAAGCTTCTGGCTCAACCTGGAGCGTGCATTACTCAAGATTCAGGATAAAAGGGAGAGCTTGGAGGTGGCC

297 L T L D V L K H G K R F H A T V S F D T D T G L K

976 CTTACTCTTGATGTTCTGAAGCATGGCAAGCGCTTCCATGCCACTGTTTCTTTTGATACAGACACAGGCCTCAAA

322 Q A L A T V N D Y A P L M K D F P L N D L L A A T

1051 CAAGCTCTGGCCACAGTGAATGACTATGCTCCCCTCATGAAGGACTTCCCACTCAATGATCTTCTGGCAGCCACG

347 E L E K I R S A V Q A I F N H L R K I R S T K Y P

1126 GAGCTGGAGAAGATCCGCTCTGCTGTGCAGGCCATCTTCAACCACCTGCGCAAGATCCGATCAACAAAGTACCCC

372 I Q R A L R L V E A I S R D L S S Q I L K V L G T

1201 ATCCAGCGTGCTCTCAGGCTGGTGGAGGCCATATCTAGGGACCTCTCCTCTCAGATCCTCAAGGTACTGGGAACA

397 R R L M H I A F D E F E K V M S Q C F D V F V T W

1276 CGCCGGTTGATGCATATCGCCTTTGATGAGTTTGAGAAGGTGATGAGTCAATGCTTTGATGTGTTTGTCACATGG

422 D D E Y E K L Q G L L R D I V K K K R D G Q L K M

1351 GATGATGAATATGAAAAGCTGCAGGGACTTCTCAGAGACATAGTAAAGAAGAAGCGTGATGGACAACTTAAGATG

447 V W R V S P A H K K L Q T R M E Q M R K F R R Q H

1426 GTGTGGCGTGTCTCCCCTGCCCATAAGAAACTACAAACACGAATGGAACAAATGCGCAAGTTTCGTCGCCAGCAC

472 E Q L R T V I V R V L R P T V V K T T T L A D G D

1501 GAGCAGCTTCGGACTGTGATCGTGCGTGTGCTGCGGCCAACTGTTGTAAAAACCACCACGCTGGCTGATGGAGAC

497 T A D L K R E M D A A G L N A I E E V N F A Y E N

1576 ACAGCTGACCTCAAGCGTGAGATGGATGCTGCAGGTCTCAATGCCATTGAAGAGGTGAACTTTGCCTATGAAAAT

522 V K E V D G L D T S K E G T D M W E A A L K R Y D

1651 GTGAAGGAGGTGGATGGCCTGGACACCAGCAAAGAGGGCACTGACATGTGGGAAGCTGCTCTCAAACGTTATGAT

547 E R I D R V E T R I T A R L R D Q L G T A K N A N

1726 GAGAGGATTGATCGTGTGGAGACACGCATCACAGCTCGACTCAGGGATCAGCTGGGCACTGCCAAAAATGCCAAC

572 E M F R I F S R F N A L F V R P H I R G A I R E Y

1801 GAGATGTTTCGTATTTTCTCTCGCTTCAATGCCTTGTTTGTGCGCCCCCACATCCGTGGTGCCATCCGAGAGTAT

597 Q T Q L I Q R V K D D I E S L H E K F K V R Y P Q

1876 CAGACACAGCTAATTCAAAGAGTCAAGGATGACATTGAGAGCCTTCATGAGAAATTCAAGGTCCGGTATCCCCAA

622 S K C C R M S T V R D L P P V S G S I I W A K Q I

1951 AGCAAGTGTTGCCGCATGAGCACGGTGAGGGACCTGCCGCCTGTTTCTGGCTCCATTATCTGGGCCAAGCAGATT

647 D R Q L T A Y M R R V E D V L G K G W E S H V E G

2026 GACCGGCAGCTGACAGCATACATGCGGCGTGTGGAAGATGTGCTTGGCAAAGGTTGGGAGAGCCATGTGGAGGGC

672 Q K L K G D G D S F R L K L N T Q D I F D D W A R

2101 CAGAAGCTGAAGGGTGATGGAGACTCATTCAGGCTCAAGCTCAATACCCAAGATATTTTTGATGATTGGGCCAGG

697 K V Q Q R N L G V S G R I F A I E T H R S K T G K

2176 AAGGTTCAGCAGAGGAACTTGGGTGTATCTGGCCGCATCTTTGCCATTGAGACGCACCGCTCCAAGACAGGCAAA

722 G N E C K L K V N F L P E T I T L S K E V R N L K

2251 GGCAATGAGTGCAAGCTGAAAGTCAACTTCTTGCCAGAGACCATCACTTTGTCCAAGGAAGTGCGGAATTTGAAG

747 N L G F R V P L T I V N K A H Q A N Q L Y P F A I

2326 AACCTTGGCTTCAGGGTACCACTGACCATTGTTAACAAGGCTCATCAGGCCAACCAGCTGTACCCATTTGCTATT

772 S L I E S V R T Y E R T L E K M E D K T N I L L L

2401 TCTCTCATAGAGAGTGTGCGCACCTATGAACGAACCCTGGAGAAGATGGAAGACAAGACTAACATCTTGTTGCTT

797 V A G M R K E V Q G L V S E G M S L V W E S Y K L

2476 GTGGCAGGCATGCGTAAAGAAGTGCAAGGACTTGTGTCTGAGGGCATGTCGCTGGTGTGGGAGTCGTACAAATTG

822 D P Y V Q R L A E C V F N F Q E K V D D S L V L E

2551 GACCCTTATGTACAACGGCTGGCAGAATGTGTCTTCAACTTCCAAGAGAAGGTTGATGACTCGCTGGTACTTGAG

847 E Q L D V D V R S L D T C Q Y A H F T F A E I L N

2626 GAACAACTGGACGTGGATGTGCGGTCATTGGACACTTGTCAGTATGCCCATTTCACCTTTGCCGAGATCCTCAAC

872 K I Q K A V D D L S L H Q Y S N L H I W V Q R L D

2701 AAGATCCAGAAAGCTGTGGATGACCTCTCTCTCCACCAGTACTCCAATCTACACATCTGGGTACAGAGGCTGGAT

897 E Q V E K K L A A R L Q A G L Q A W T A A L L G Q

2776 GAACAGGTTGAGAAGAAGCTTGCAGCACGTCTTCAGGCCGGCCTCCAGGCATGGACAGCAGCTCTTCTAGGACAG

922 Q Q K D E D I D I P M D T D T P D K P T H K P G G

2851 CAGCAGAAGGATGAGGATATTGACATTCCTATGGACACTGATACTCCTGATAAACCAACACACAAGCCTGGTGGA

947 A P K I N Q V V Q E V R I T N Q T M Y M H P A I E

2926 GCTCCAAAGATCAACCAGGTGGTGCAAGAGGTGCGCATCACCAACCAGACCATGTACATGCACCCAGCCATAGAG

972 Q A R Y Q I M E Q L F G W Q A I V T S Q N R I Q S

3001 CAGGCACGCTACCAGATCATGGAGCAACTCTTCGGCTGGCAGGCCATTGTCACCTCACAGAACCGCATCCAGAGC

997 T R Y Q V G L D R P T T Q V Y K N L L T K L P E R

3076 ACCCGATACCAGGTTGGACTGGACCGACCCACCACCCAAGTCTATAAAAATCTGCTCACCAAGCTCCCTGAGAGG

1022 H T Y I K A A Y K A I E M V V D E V T T Y V Q E W

3151 CACACCTACATCAAGGCTGCTTACAAGGCAATTGAGATGGTAGTGGATGAAGTGACCACTTATGTCCAAGAGTGG

1047 L Q Y Q A L W D L Q P D H L Y G Q L G D D V A K W

3226 CTGCAGTATCAAGCTCTGTGGGATTTGCAGCCAGATCATCTTTATGGCCAGCTGGGTGATGATGTGGCCAAGTGG

1072 M K L L Q D I K Q C R T T F D T S D T R R G F G P

3301 ATGAAGCTTCTACAGGACATCAAACAGTGCCGCACCACATTTGACACCTCAGACACTCGGCGCGGCTTTGGACCC

1097 I V V D Y A K V Q S K V S L K Y D S W H K D V L S

3376 ATTGTTGTGGACTATGCCAAGGTGCAGAGCAAAGTGTCCCTCAAGTATGACTCGTGGCACAAGGATGTCCTCAGC

1122 K F G Q L L G Q E M T Q F H A Q V S K S R S E L E

3451 AAGTTTGGACAGCTGCTGGGTCAGGAGATGACACAGTTCCATGCTCAGGTGTCAAAGAGCCGAAGTGAGCTGGAG

1147 Q Q S I D A A S T S D A V T L I T Y V Q A L K R K

3526 CAGCAGAGCATTGATGCGGCCAGCACCTCTGATGCTGTGACCCTCATCACTTATGTACAAGCTCTTAAACGCAAG

1172 M K S W E K H V E M Y K E G Q R I L E R Q R F Q F

3601 ATGAAATCATGGGAGAAGCATGTTGAAATGTACAAGGAGGGACAACGGATCTTGGAGCGGCAACGCTTCCAGTTT

1197 P N Q W L H V D N I E G E W G A F N E I I R R K D

3676 CCTAACCAATGGTTGCATGTGGACAACATTGAGGGTGAATGGGGAGCCTTCAACGAGATCATCCGCCGCAAAGAT

1222 S S I Q T Q V A S L Q L K I V A E D K A V E S R T

3751 TCAAGCATCCAGACCCAGGTGGCCTCCCTGCAACTTAAGATTGTAGCAGAGGACAAGGCTGTAGAGTCCCGCACC

1247 N D F L Q D W E H G K P V E G H L R P D E A L Q R

3826 AATGACTTCCTGCAGGACTGGGAGCATGGCAAGCCAGTGGAAGGACACCTGCGACCAGATGAGGCATTGCAGCGC

1272 L T L Y E S K F T R L K D D R D N V A K A K E A L

3901 CTTACCCTGTATGAAAGCAAGTTCACCCGTCTCAAGGATGACCGAGACAATGTGGCCAAGGCCAAGGAAGCTTTG

1297 E L Q E P G A L N T S E D R M T V A Y E E L Q D L

3976 GAGCTCCAGGAGCCTGGAGCCCTCAACACATCAGAAGACAGAATGACAGTGGCCTATGAAGAGCTGCAGGACCTG

1322 K G V W A E L A K I W E Q I D E M K E K P W L S V

4051 AAGGGAGTGTGGGCTGAGCTGGCCAAGATCTGGGAGCAGATTGATGAGATGAAGGAGAAGCCTTGGCTCTCAGTG

1347 Q P R K L R G Q L D Q L L T Q L K D L P A R L R Q

4126 CAACCCAGAAAACTACGTGGCCAGCTAGATCAGCTGTTGACTCAATTGAAGGACTTGCCAGCCCGCCTGCGTCAG

1372 H A S Y E Y V K R L L Q G Y T K V N M T I I E L K

4201 CATGCAAGCTATGAGTATGTGAAACGGCTGCTGCAGGGCTACACCAAGGTGAACATGACCATTATTGAACTGAAG

1397 S D A L K E R H W K T L M K Q L H V S W L L S D L

4276 AGTGACGCCCTCAAGGAGCGCCACTGGAAGACCCTAATGAAGCAGCTGCATGTCTCCTGGTTGTTGAGTGATTTG

1422 T L G Q V W D V D L V K N E A I I K N V I I T A Q

4351 ACCTTGGGGCAGGTGTGGGATGTGGACCTGGTGAAGAATGAGGCCATTATCAAGAATGTGATCATTACTGCCCAG

1447 G E M A L E E F L K Q V R E S W Q C Y E L D L I N

4426 GGTGAGATGGCCCTGGAAGAGTTCCTGAAGCAGGTGAGGGAGTCCTGGCAGTGTTATGAGCTGGATCTTATCAAC

1472 Y Q N K C K L I R G W D D L F N K V K E H I N S V

4501 TACCAGAACAAGTGCAAGCTGATCCGTGGCTGGGATGATCTCTTCAACAAGGTGAAGGAGCACATCAACTCAGTG

1497 A A M K L S P Y Y R E F E E E A L T W E E K L N R

4576 GCTGCTATGAAACTGTCTCCCTACTACAGGGAGTTTGAAGAGGAGGCACTCACCTGGGAAGAGAAACTGAACAGG

1522 I N S L F D V W I D V Q R R W V Y L E G I F S G S

4651 ATTAACTCCCTCTTTGATGTATGGATTGACGTGCAGAGGCGCTGGGTCTACTTGGAGGGGATCTTCTCTGGGTCC

1547 S D I K A L L P V E T S R F S S I S T E F L N L M

4726 TCCGACATCAAGGCTCTGCTGCCTGTGGAGACCTCACGCTTCAGCAGCATTAGTACTGAATTCTTGAACCTGATG

1572 K K V S K S P M V M D V L N I Q G V Q R S L E R L

4801 AAGAAGGTCAGCAAATCACCGATGGTGATGGATGTACTCAACATTCAGGGTGTCCAGCGATCCCTGGAGCGCCTG

1597 A D L L G K I Q K A L G E Y L E R E R S S F P R F

4876 GCTGATCTGCTGGGCAAAATACAGAAGGCCCTTGGCGAGTACTTGGAGCGAGAGAGATCTTCCTTCCCCAGATTC

1622 Y F V G D E D L L G I I G N S K N I P R L Q K H F

4951 TATTTTGTGGGAGATGAGGACTTGCTGGGGATTATTGGCAATAGCAAGAACATTCCTCGGCTTCAGAAACACTTC

1647 K K M F A G V T A I N L D E T E T V V T G I A S R

5026 AAGAAGATGTTTGCTGGGGTGACTGCTATCAATCTGGATGAGACAGAGACTGTAGTCACTGGGATTGCCTCCAGG

1672 E G E E V P F N K V V S T V D F P K I N E W L A Q

5101 GAGGGAGAAGAGGTACCATTCAACAAAGTGGTATCAACAGTGGACTTCCCCAAGATCAATGAATGGCTGGCCCAG

1697 V E K E M R V T L A Q N L A A A V G E I K Q F K H

5176 GTAGAGAAGGAGATGAGGGTGACACTGGCTCAGAACTTGGCAGCTGCAGTGGGAGAGATCAAGCAATTCAAGCAT

1722 G P I D T A S Y L A W C D K Y Q A Q L V V L A A Q

5251 GGCCCCATTGACACAGCCAGCTACTTAGCCTGGTGTGACAAGTACCAAGCCCAGCTGGTTGTGCTGGCTGCCCAG

1747 I S W S E E V E A G L V A A S N G D S G P L D Q T

5326 ATTTCATGGAGTGAAGAGGTTGAAGCTGGCCTGGTAGCAGCATCTAATGGAGACTCTGGACCGCTGGATCAGACC

1772 L V Q V E A T L N V L A D S V L Q E Q P L L R R K

5401 TTGGTTCAGGTGGAGGCCACTCTTAATGTGTTGGCTGACTCAGTCCTACAGGAGCAACCTCTCTTGCGCCGCAAA

1797 K L E H L I S G F V H K R T V T R N L I K N K I T

5476 AAGCTGGAACACCTGATCAGTGGGTTTGTCCACAAGAGGACAGTGACCAGAAACCTAATCAAGAACAAGATAACC

1822 N P K S F D W L S Q M R F Y F D P K Q S D P L K Q

5551 AACCCTAAGTCCTTTGATTGGTTGAGCCAAATGAGATTCTACTTTGACCCAAAGCAGAGTGATCCCTTGAAGCAG

1847 L S I H M A N A K F N Y G F E Y L G V Q D K L V Q

5626 CTCAGCATCCACATGGCCAATGCCAAGTTTAACTATGGCTTTGAGTACCTTGGCGTACAGGACAAGCTGGTACAG

1872 T P L T D R S Y L T M T Q A L E A R L G G S P F G

5701 ACACCTCTCACTGACCGCAGCTACCTCACCATGACACAGGCGCTGGAGGCTCGTCTTGGAGGCTCACCGTTTGGT

1897 P A G T G K T E S V K A L G N Q L G R F V L V F N

5776 CCAGCTGGTACTGGCAAGACTGAGTCTGTCAAGGCTCTGGGCAACCAGCTGGGTCGCTTTGTGCTTGTGTTCAAC

1922 C D E T F D F Q A M G R I F V G L C Q V G A W G C

5851 TGTGATGAGACCTTTGACTTCCAGGCCATGGGCAGAATTTTTGTTGGTCTTTGCCAGGTAGGGGCATGGGGATGC

1947 F D E F N R L E E R M L S A V S Q Q I Q T I Q E A

5926 TTTGACGAGTTCAACCGTCTGGAGGAGAGAATGCTGTCGGCTGTGTCCCAGCAGATCCAGACCATCCAGGAGGCC

1972 L K E K A G T N S N T S V G Q V L T V E L V G K Q

6001 CTCAAGGAGAAGGCTGGCACCAACAGCAACACGAGTGTTGGTCAGGTGTTGACCGTGGAGCTGGTGGGCAAGCAA

1997 V K V S T D M A I F I T M N P G Y A G R S N L P D

6076 GTGAAGGTATCCACTGACATGGCCATCTTCATCACCATGAACCCTGGATATGCTGGCCGTTCTAATCTCCCAGAT

2022 N L K K L F R S L A M T K P D R Q L I A E V M L F

6151 AACCTTAAGAAGCTGTTCCGCTCCCTGGCTATGACTAAGCCTGACCGACAGCTCATTGCAGAGGTCATGCTGTTC

2047 S Q G F R S A E K L A S K I V P F S K L C D E Q L

6226 AGTCAAGGCTTCAGATCAGCTGAGAAGCTAGCCAGCAAAATTGTTCCATTCTCCAAACTTTGTGATGAGCAGCTG

2072 S N Q S R Y D F G L R A L K S V L V S A G N V K R

6301 TCCAACCAGTCCCGCTATGACTTTGGTCTGCGTGCCCTCAAGTCAGTGTTGGTGTCTGCTGGCAATGTGAAGCGT

2097 D R I Q R I K E D L R S R E E A V I D E G T I A E

6376 GATCGCATTCAGCGCATCAAGGAGGACCTCAGGAGCAGGGAGGAGGCAGTCATTGATGAGGGCACTATTGCAGAG

2122 N L P E Q E I L I Q S V C E T M V P K L V A E D I

6451 AATCTTCCAGAGCAGGAGATCCTGATTCAGAGTGTATGTGAGACAATGGTGCCCAAGCTCGTGGCAGAGGACATC

2147 P L L F S L L S D V F P G I E Y T R A Q M S G L K

6526 CCCCTGTTGTTCTCCCTACTGTCTGATGTGTTCCCAGGCATTGAGTACACACGTGCCCAGATGTCTGGCCTTAAA

2172 E E I R K V C R E Q L L V C G E G E E Q G A H W M

6601 GAAGAGATCAGGAAGGTGTGCAGGGAGCAGCTCCTGGTGTGTGGTGAGGGGGAGGAGCAGGGGGCACACTGGATG

2197 E K V L Q P Y Q I S N L N H G L M M V G P S G S G

6676 GAGAAGGTGCTCCAGCCGTACCAGATCAGCAACCTTAACCATGGTCTTATGATGGTGGGGCCCAGTGGCTCAGGC

2222 K S T A W R V L L K A L E R F E G V E G V A H V I

6751 AAGTCAACAGCCTGGCGTGTGCTGCTGAAGGCCCTGGAGCGCTTTGAGGGAGTGGAGGGTGTGGCACATGTCATT

2247 D P K A M S K E A L Y G V L D P N T R E W T D G L

6826 GACCCCAAGGCCATGAGCAAGGAAGCACTTTATGGTGTGCTGGACCCCAACACCCGGGAGTGGACTGACGGCCTC

2272 F T H I L R K I I D N V R G E I N K R Q G I I F D

6901 TTCACACACATCCTCAGGAAGATCATTGACAATGTTCGTGGAGAAATCAATAAACGGCAGGGGATTATCTTTGAT

2297 G D V D P E W V E N L N S V L D D N K L L T L P N

6976 GGAGATGTGGATCCTGAGTGGGTGGAGAACCTTAACTCAGTGCTGGATGACAACAAGCTACTCACGCTGCCCAAT

2322 G E R L S I P P N V R I M F E V Q D L K Y A T L A

7051 GGCGAGCGTCTCTCTATCCCGCCAAATGTCCGGATCATGTTTGAGGTGCAAGACCTGAAGTATGCCACTCTGGCT

2347 T V S R C G M V W F Y E D V L S V E M I F E N Y L

7126 ACTGTGTCTCGCTGTGGTATGGTATGGTTCTATGAGGATGTGCTGTCTGTGGAGATGATCTTTGAGAACTACCTG

2372 S R L S N I P V E E T E E D I A F I K K T D K K T

7201 TCCCGCCTGTCTAACATCCCGGTGGAGGAGACTGAAGAAGACATTGCTTTCATCAAGAAAACAGACAAGAAGACT

2397 D D E I T P H C S A E R C G F H P V T T S H P R W

7276 GATGATGAGATTACCCCACACTGCAGTGCAGAGAGATGCGGCTTCCATCCTGTCACCACATCTCACCCCAGATGG

2422 P C V K C L E Y A T N N L E H I M D F T R M R A L

7351 CCTTGTGTGAAGTGCTTGGAATATGCCACCAACAACCTTGAACACATCATGGACTTCACCCGTATGAGGGCACTC

2447 S S L F A L L N Q A V R N I V Q Y N N T H P D F P

7426 TCATCCCTCTTTGCCCTCCTCAACCAGGCTGTGAGGAATATTGTGCAGTACAACAATACCCACCCTGACTTCCCC

2472 M H T D Q L E R Y V P K A L V H S L L W S F A E D

7501 ATGCACACAGATCAGCTGGAGAGGTATGTCCCTAAGGCACTGGTGCACTCATTGTTGTGGAGCTTTGCTGAGGAT

2497 G K L K A R S D M G D F I R N S T T I P L P P N T

7576 GGCAAGCTGAAGGCTCGCAGTGACATGGGCGACTTCATCCGTAACTCCACCACCATCCCACTGCCACCAAACACC

2522 S N P I I D Y E V S I S G E W M L W S A K V P S M

7651 AGTAATCCAATCATTGACTATGAGGTGTCCATCAGTGGAGAGTGGATGTTGTGGTCAGCCAAGGTGCCCAGCATG

2547 E V E T H K V A T P D V V V P T V D T V R H E A L

7726 GAGGTGGAGACTCACAAAGTGGCAACACCTGATGTAGTGGTGCCCACCGTGGACACAGTGCGCCATGAGGCTCTC

2572 L Y T W L A E H K P L V L C G P P G S G K T M T L

7801 CTCTACACCTGGCTAGCTGAACACAAACCTCTTGTGCTGTGTGGCCCACCTGGCTCAGGCAAGACTATGACCTTG

2597 F S A L R A L P D L E V V G L N F S S A T T P E L

7876 TTCTCTGCCCTGCGAGCCCTGCCAGATTTGGAAGTGGTGGGCCTTAACTTCTCCAGTGCTACCACACCAGAGCTC

2622 L L K T F D H Y C E Y R K T P N G I I L S P V Q L

7951 CTGCTGAAGACCTTTGACCACTACTGCGAGTACCGTAAGACTCCCAATGGCATTATCCTGTCCCCTGTGCAGCTG

2647 G K W L V V F C D E I N L P D M D K Y G T Q R V I

8026 GGCAAGTGGCTGGTGGTATTCTGTGACGAGATCAACTTGCCGGATATGGACAAGTACGGCACTCAGCGTGTCATC

2672 S F L R Q M V E H G G F F R I S D Q A W V K L E R

8101 AGCTTCCTTCGGCAGATGGTGGAGCACGGTGGCTTCTTCAGGATCTCAGACCAAGCATGGGTGAAGCTGGAACGC

2697 I Q F V G A C N P P T D P G R K P L S H R F L R H

8176 ATCCAGTTTGTGGGTGCCTGTAATCCTCCAACTGATCCTGGCCGAAAGCCCCTCTCCCACCGGTTCCTGCGCCAT

2722 V P V V Y V D Y P G E T S L K Q T Y G S F N R A M

8251 GTACCAGTGGTGTATGTGGACTACCCTGGAGAGACATCCCTAAAGCAGACCTATGGTTCCTTCAACAGGGCCATG

2747 L R I I P P L R S Y A D P L T N A M V E F Y L Q S

8326 CTCAGGATCATTCCTCCTCTCAGATCATATGCAGACCCACTGACCAATGCCATGGTGGAGTTCTACCTCCAATCT

2772 Q E R F T Q D M Q P H Y V Y S P R E M T R W V R G

8401 CAGGAGCGCTTCACACAGGACATGCAGCCGCACTATGTGTACTCTCCACGTGAAATGACGAGATGGGTGCGAGGC

2797 I C E A I R P L E T L D V E G L V R L W A H E A L

8476 ATCTGTGAGGCCATCAGGCCATTGGAAACCCTGGATGTTGAGGGACTGGTCAGACTATGGGCCCATGAGGCTCTC

2822 R L F H D R L V T D E E R K W T N D N V D A I A L

8551 CGGCTTTTCCATGACCGTCTGGTAACAGATGAGGAGCGCAAGTGGACCAATGACAATGTGGATGCTATTGCTCTC

2847 K H F P N T D R D R A L H R P I L Y S N W L S K D

8626 AAGCACTTCCCCAACACTGATCGAGACAGAGCTCTGCACCGACCCATCTTGTACTCAAACTGGCTGTCCAAGGAC

2872 Y L P V D Q E E L R E Y V K A R L K V F Y E E L

8701 TATCTTCCAGTAGACCAGGAGGAGCTGAGGGAATATGTTAAGGCCAGGCTGAAGGTGTTCTACGAGGAGGAGCTA

2897 D V P L V L F N E V L D H V L R I D R I F R Q P Q

8776 GATGTGCCCCTGGTGTTGTTTAACGAGGTGCTTGACCACGTGCTGCGTATTGACCGTATCTTCCGGCAGCCTCAG

2922 G H L L L I G A S G A G K T T L S R F V A W M N G

8851 GGTCACTTGCTGCTTATTGGAGCCTCAGGGGCTGGCAAGACCACCTTGTCACGCTTTGTGGCTTGGATGAATGGC

2947 L S I F Q I K V H N K Y T S E D F D E D L R A V L

8926 CTCTCCATCTTCCAGATCAAAGTGCACAACAAATACACTTCAGAAGACTTCGATGAGGACCTGCGAGCAGTGCTC

2972 R R S G C K D E K I C F I L D E S N V L D S S F L

9001 CGGCGCTCAGGCTGCAAGGATGAGAAAATATGCTTCATTTTGGATGAGTCCAATGTGTTGGATTCCTCCTTCTTG

2997 E R M N T L L A N G E V P G L F E G D E Y T T L M

9076 GAACGCATGAACACCCTGCTGGCCAACGGTGAGGTGCCAGGTCTGTTTGAGGGTGACGAGTACACTACCCTCATG

3022 T Q C K E G S Q R E G L M L D S S E E L Y K W F T

9151 ACCCAGTGCAAAGAAGGCAGCCAGCGTGAAGGGCTCATGTTGGATTCCAGTGAGGAGCTGTACAAGTGGTTCACT

3047 G Q V I R N L H V V F T M N P S A D G L K D R A A

9226 GGCCAGGTTATAAGGAACCTTCATGTGGTGTTCACCATGAATCCCTCTGCTGATGGTCTCAAGGACCGTGCTGCA

3072 T S P A L F N R C V L N W F G D W S N G A L F Q V

9301 ACCTCCCCTGCCTTGTTCAACAGGTGTGTCCTCAACTGGTTTGGAGACTGGTCCAATGGTGCCCTGTTCCAGGTA

3097 G M E F T H R I D L D R M N W A A P D Y F P V A Y

9376 GGAATGGAGTTCACGCACCGCATTGATCTGGACAGAATGAACTGGGCAGCCCCAGACTACTTCCCAGTAGCTTAT

3122 E C L P A P P S H R D A I I N A F V H V H Q T L H

9451 GAGTGCCTGCCTGCGCCGCCTAGCCACAGGGATGCCATCATCAATGCCTTTGTACATGTGCATCAGACTCTGCAC

3147 R A N T R L A K R G S T V M A I T P R H Y L D F I

9526 CGAGCCAACACTCGTCTGGCCAAGAGAGGCAGCACTGTTATGGCCATCACTCCAAGACACTACCTGGACTTCATC

3172 D H F V K L Y N E K R S E L E E Q Q L H L N V G L

9601 GATCACTTTGTGAAGCTGTACAATGAAAAACGATCAGAGCTTGAGGAGCAGCAACTCCATCTGAATGTAGGTCTG

3197 G K I A E T V E Q V E Q M Q K S L A I K N Q E L Q

9676 GGCAAGATTGCCGAAACAGTGGAGCAGGTGGAACAGATGCAGAAATCCTTGGCCATCAAAAACCAGGAGCTGCAA

3222 A K N E A A N L K L K Q M V K D Q Q E A E K Q K V

9751 GCCAAGAATGAAGCAGCCAATCTTAAGCTGAAACAGATGGTGAAGGATCAACAAGAGGCTGAGAAGCAGAAGGTT

3247 A S Q E L Q E Q L E A Q T V R I T I K R E E V M Q

9826 GCCAGTCAAGAGCTGCAGGAGCAGCTAGAAGCTCAAACTGTACGTATCACAATCAAGCGAGAGGAGGTGATGCAG

3272 D L S K V E P A V Q D A Q N A V K S I K E Q H L V

9901 GATCTGAGCAAAGTAGAACCAGCAGTGCAGGATGCCCAGAATGCTGTGAAGAGCATCAAAGAGCAGCATCTGGTG

3297 E V R S M A N P P P M V K L A L E S I C L L L G E

9976 GAGGTGAGGTCTATGGCCAACCCTCCTCCCATGGTCAAGTTGGCCCTGGAATCCATCTGTTTGTTGCTGGGAGAA

3322 A T S D W K T I K S I I M K D N F I G T V V N Y N

10051 GCCACCTCCGATTGGAAGACCATCAAATCCATCATCATGAAGGATAATTTCATTGGCACAGTTGTCAACTACAAC

3347 T D D M P D D I R E K M R N K Y L S N P D Y N F E

10126 ACAGATGACATGCCTGATGATATCCGTGAGAAGATGCGCAACAAGTATCTCAGCAACCCTGATTATAACTTTGAG

3372 K V N R A S L A C G P M V K W A I A Q I E F A D M

10201 AAGGTGAACCGTGCTTCACTGGCCTGTGGACCTATGGTCAAGTGGGCCATTGCTCAAATAGAATTTGCTGACATG

3397 L K R V E P L R N E L S S L E H M A E E N K K K H

10276 TTGAAGAGAGTGGAACCTCTGAGAAATGAACTGAGCAGTCTTGAACACATGGCCGAAGAGAACAAGAAGAAGCAT

3422 E D V K G L I T R L E R S I A A Y K D E Y A L L I

10351 GAAGATGTGAAGGGTCTCATTACACGTCTGGAACGTTCCATTGCTGCTTACAAAGATGAATATGCACTGCTCATA

3447 S Q A Q A I K T D L E N V Q A K V D R S M A L I R

10426 TCTCAGGCCCAGGCCATCAAGACTGACCTGGAGAATGTGCAGGCCAAGGTGGACCGATCCATGGCCCTCATCAGG

3472 N L S I E K D R W A A T S D T F K F Q M S T I I G

10501 AACCTCAGCATCGAGAAGGACCGCTGGGCAGCCACCTCAGACACCTTCAAGTTCCAGATGTCCACCATCATTGGA

3497 D V L L P S A F L A Y G G Y F D Q Q F R E N L F S

10576 GATGTGCTACTTCCTTCTGCTTTCCTAGCCTATGGTGGCTACTTTGACCAGCAGTTCAGAGAGAATCTGTTCAGC

3522 N W C H H L L Q A N L Q F R G D I A R T E Y L S N

10651 AACTGGTGTCACCATCTGCTGCAGGCCAACCTTCAATTCCGAGGAGACATTGCCCGCACTGAGTACCTGTCCAAC

3547 P D E R L R W Q A N A L P A D E L C T E N A I R L

10726 CCTGATGAAAGGCTGCGCTGGCAAGCAAATGCCCTTCCTGCTGACGAGCTCTGCACTGAGAATGCCATCAGGCTC

3572 N R F N R Y P L I I D P S G Q A T E F I M N E F K

10801 AACAGGTTCAACAGATACCCACTCATCATTGATCCCTCAGGCCAGGCCACAGAGTTCATCATGAATGAATTCAAA

3597 D K K I T K T S F L D D S F R K N L E S A L R F G

10876 GACAAGAAAATCACAAAGACCAGCTTCCTGGATGACTCTTTCAGAAAGAACCTTGAATCAGCCCTTAGGTTTGGC

3622 N P L L V Q D V E N Y D P I L N P V L N R E L R R

10951 AATCCACTGCTGGTACAAGATGTTGAGAATTACGATCCCATCCTGAACCCTGTGCTGAACCGTGAACTGCGACGT

3647 T G G R V L I T L G D Q D I D L S P A F T I F L S

11026 ACTGGTGGTCGTGTTCTCATCACCCTTGGAGACCAGGACATTGATCTTTCTCCAGCTTTCACCATCTTCCTTTCT

3672 T R D P T V E F P P D I C S R V T F V N F T V T R

11101 ACTCGTGACCCTACAGTAGAGTTCCCACCAGACATCTGTTCCCGTGTCACTTTTGTCAACTTCACAGTAACACGG

3697 S S L Q S Q C L N Q V L K A E R P D I D D K R S N

11176 TCATCTCTGCAGTCCCAGTGTCTTAACCAAGTCCTCAAGGCTGAGCGGCCAGACATTGATGACAAGCGGTCCAAC

3722 L L K L Q G E F Q L R L R Q L E K N L L Q V L S D

11251 CTGCTCAAGCTTCAGGGTGAATTCCAGTTGCGTCTGAGACAGCTAGAGAAGAACCTGCTGCAAGTACTGAGTGAC

3747 S K G Q I L D D D S V I T T L E T L K E E A A D I

11326 TCCAAGGGGCAGATCCTGGATGATGACTCCGTGATAACCACCCTTGAGACCTTGAAGGAGGAGGCGGCAGACATT

3772 S L K V Q E T D K T M N E I E M V S Q Q Y L P L S

11401 TCCCTCAAGGTGCAAGAGACTGACAAGACAATGAATGAGATTGAGATGGTGTCACAGCAGTACTTGCCACTCTCA

3797 L A C S S I Y F T L D S L N Q I H F L Y Q Y S L H

11476 TTGGCCTGCTCCAGTATCTACTTCACACTCGACTCACTGAACCAGATCCACTTCTTATACCAGTATTCTCTGCAC

3822 F F L D V F T D V L S N N P N I R D H T D Y S R R

11551 TTCTTCCTGGACGTGTTCACCGATGTGCTCAGCAACAATCCCAACATCCGGGACCACACTGACTACTCTCGCCGC

3847 L S I I T N D L F Q L T Y T R V A R G M L H T D R

11626 CTGTCCATCATCACCAACGACCTCTTCCAGTTGACATATACCAGAGTGGCCCGAGGAATGCTGCACACGGACCGC

3872 L V L A V L L A R I R L R G V T S E P S Y D T E F

11701 CTGGTGCTGGCTGTTCTGCTTGCCCGCATCCGCCTGAGGGGAGTCACCTCAGAACCTTCCTATGACACAGAGTTC

3897 Q F L L K S K E G L L N T T V S A T A G L T P E Q

11776 CAGTTCCTCCTGAAGAGCAAAGAAGGTCTTCTCAACACCACAGTCTCAGCCACGGCTGGTCTTACCCCAGAGCAG

3922 T D S M L R L C N K V P A F A N M D R L V G N K D

11851 ACAGACTCCATGCTGAGGCTTTGTAACAAGGTACCAGCCTTTGCCAATATGGATCGCCTAGTGGGGAACAAAGAT

3947 F Q A W L E S A A P E L N V P T I W E T E K Q L S

11926 TTCCAGGCTTGGCTTGAGTCTGCCGCCCCAGAGCTGAATGTGCCAACCATCTGGGAGACAGAGAAGCAACTGTCA

3972 P V G Q S M Y H I L A I Q A F R P D R L Y A A A G

12001 CCAGTCGGCCAGTCTATGTACCACATTCTGGCCATCCAGGCATTCAGACCAGACAGATTGTATGCAGCTGCTGGG

3997 L F V N N V L G D G F L G I G E R E V N L A S V V

12076 CTGTTTGTTAACAATGTGTTGGGTGATGGCTTCTTGGGCATTGGTGAGCGGGAGGTGAACTTGGCATCAGTGGTG

4022 E G E V K A N T P V L M C A A Q G F D A S V R V D

12151 GAGGGTGAAGTGAAGGCCAACACTCCAGTGCTCATGTGTGCTGCCCAGGGCTTTGATGCCTCAGTCAGGGTTGAT

4047 N L A A E L S K G M T S I A F G S A E G F S Q A E

12226 AACTTGGCAGCAGAACTCAGCAAAGGAATGACCTCCATTGCCTTTGGCTCAGCAGAAGGTTTCAGCCAGGCTGAG

4072 K A I N S A V K N G R W V M L K N V H L A P A W L

12301 AAAGCCATCAACTCGGCCGTCAAGAATGGAAGGTGGGTGATGTTGAAGAATGTCCACCTGGCACCTGCCTGGTTG

4097 V Q L E K K L H S L H P H P Q F R L F L T M E I T

12376 GTCCAACTAGAGAAGAAGCTCCACTCCCTTCACCCACATCCACAATTCCGGCTCTTCCTCACCATGGAGATCACA

4122 P R L P V N L L R A G R V F V F E P P P G V R A N

12451 CCACGCCTGCCTGTCAACCTCCTGCGTGCTGGCCGTGTGTTTGTGTTTGAGCCGCCACCAGGCGTCCGTGCCAAC

4147 L L R T F S T V P A S R M M R S P N E R A R L Y F

12526 CTGCTGCGAACTTTCAGCACGGTGCCGGCCTCAAGGATGATGCGGTCGCCCAATGAGCGTGCCCGCCTCTACTTC

4172 L L A W F H A I T Q E R L R Y A P L G W A E Y Y E

12601 CTGCTGGCTTGGTTCCACGCCATCACACAGGAACGGCTGCGTTACGCTCCACTTGGCTGGGCCGAGTACTATGAA

4197 F N E S D L R V A C D T L D T W I G A T A M G R T

12676 TTCAATGAGTCAGATCTGCGTGTGGCCTGTGACACTCTCGACACCTGGATTGGAGCCACTGCTATGGGTCGAACA

4222 N L P P E K V P W D A L A T L M S Q C I Y G G K I

12751 AATCTGCCTCCAGAGAAAGTACCATGGGATGCCTTGGCCACCCTCATGTCCCAGTGCATTTATGGTGGCAAGATT

4247 D D E F D Q R L L T S F L N K L F T P K S F D P E

12826 GACGATGAATTTGACCAAAGGCTGCTCACTTCCTTCCTCAACAAGCTGTTCACTCCCAAGAGCTTTGACCCTGAG

4272 F P L V C N V D G V E N Q N I T M P D G I R R D Q

12901 TTCCCTCTGGTCTGCAATGTAGACGGTGTTGAAAACCAGAACATCACCATGCCTGATGGAATCCGACGTGACCAG

4297 F M Q W V E S L T D R Q T P S W L G L P N N A E K

12976 TTCATGCAGTGGGTGGAGTCCCTCACTGATCGCCAGACTCCCAGTTGGCTTGGGCTGCCCAACAATGCTGAGAAG

4322 V L L T T R G T D M L S K L M K M Q V L E G D E E

13051 GTCCTGCTCACCACCAGGGGCACAGACATGTTGAGCAAACTGATGAAAATGCAAGTTCTTGAGGGTGATGAGGAG

4347 L A Y G G D G T P G T E R K D I E G R P S W M V T

13126 CTGGCCTATGGCGGGGATGGCACACCTGGCACAGAGCGGAAAGATATTGAGGGCCGTCCATCGTGGATGGTAACA

4372 L H Q S A V T W L S H L P E S V A N L R R T M D N

```
13201 TTGCACCAGTCTGCTGTCACCTGGCTTTCACACCTGCCTGAGTCAGTGGCTAATCTGCGTCGTACAATGGACAAC
 4397 I  K  D  P  L  Y  R  Y  F  E  R  E  V  N  S  G  A  K  L  L  R  V  V  K  S
13276 ATCAAGGATCCACTGTATCGTTACTTTGAACGGGAAGTGAACTCTGGTGCTAAATTGTTGCGTGTGGTGAAGAGT
 4422 D  L  N  D  V  V  L  I  C  K  A  E  K  K  Q  T  N  Y  H  R  N  M  V  S  Q
13351 GACCTCAATGATGTTGTCCTCATCTGCAAGGCTGAGAAGAAACAGACAAATTACCACCGTAATATGGTGAGCCAG
 4447 L  V  K  G  I  I  P  D  Q  W  K  A  Y  T  V  P  R  G  A  T  V  I  Q  W  I
13426 CTAGTGAAAGGCATCATTCCTGACCAGTGGAAGGCTTACACAGTGCCTCGTGGAGCCACAGTCATCCAGTGGATC
 4472 T  D  F  S  Q  R  V  K  Q  L  Q  E  I  S  Q  Q  V  A  Q  F  G  A  G  E  L
13501 ACAGACTTCTCCCAGCGTGTCAAACAGCTGCAGGAGATTTCACAGCAAGTGGCTCAGTTTGGAGCAGGAGAATTG
 4497 K  N  V  T  V  W  L  G  G  L  F  N  P  E  A  Y  V  T  A  T  R  Q  C  V  A
13576 AAGAATGTAACGGTGTGGCTGGGAGGTCTGTTCAACCCGGAAGCCTACGTCACTGCAACACGGCAGTGTGTAGCC
 4522 Q  A  N  S  W  S  L  E  E  L  M  L  E  V  S  I  A  D  A  E  G  Q  I  T  A
13651 CAGGCCAACTCTTGGTCCTTGGAGGAACTAATGTTAGAAGTATCCATTGCTGATGCTGAGGGCCAGATCACTGCT
 4547 A  G  G  F  S  I  K  G  L  K  L  Q  G  A  V  C  V  S  N  A  L  Q  L  T  H
13726 GCAGGTGGCTTTTCTATCAAGGGACTAAAGCTGCAGGGAGCAGTGTGTGTGAGTAATGCACTGCAGTTGACCCAT
 4572 T  I  M  T  D  L  P  M  V  T  L  H  W  V  R  R  E  P  V  D  T  N  K  I  T
13801 ACCATCATGACTGACCTACCGATGGTAACGCTCCACTGGGTACGTCGGGAACCTGTTGATACTAACAAGATCACA
 4597 L  P  V  Y  L  N  A  T  R  T  D  I  L  F  T  I  D  L  K  I  A  A  N  E  N
13876 TTGCCAGTGTACCTCAATGCCACCCGCACAGACATCCTCTTCACAATCGACCTTAAAATTGCAGCAAATGAGAAT
 4622 A  H  S  F  Y  E  R  G  V  A  L  L  A  S  I  S  L  N  *
13951 GCACACAGTTTCTATGAGAGGGGTGTGGCACTTCTTGCTTCTATCAGTCTCAATTAAaggatctgtaaagacacc
14026 acagacaagctttaagttattgacagtaccgttacaattttgtcaagatcatcaatgatgtttggagtaattaag
14101 gctatttaaagtatttatcttgtagtcaggctcttaataatatagaaggaaaatgttaattaataaaaagagcag
14176 tgattgaaaaacaataaaatacactataacataaaaaaaaaaaa
```

Figure S1： Nucleic acid sequence and translated protein sequence of *Pt-dhc*.

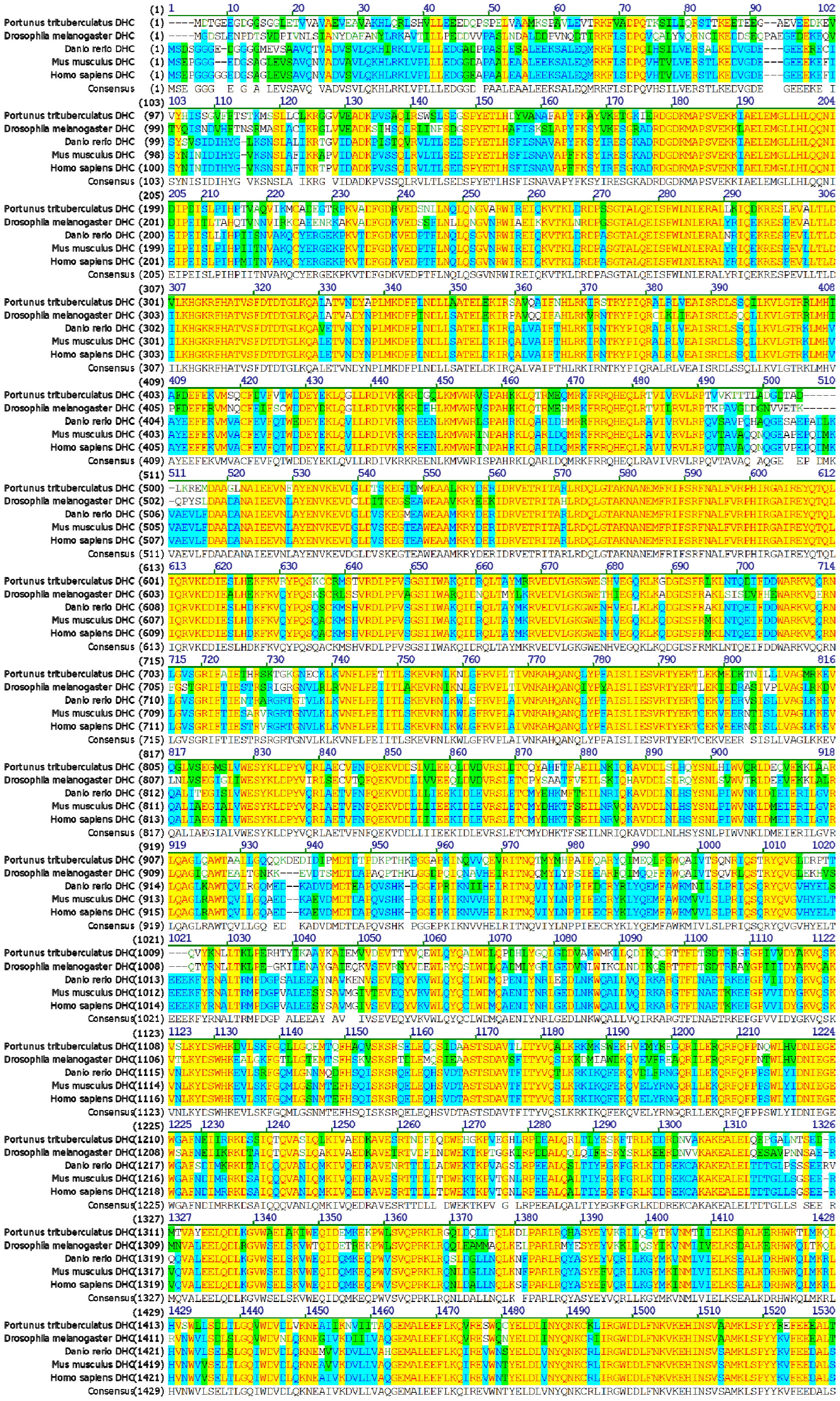
Portunus trituberculatus DHC
Drosophila melanogaster DHC
Danio rerio DHC
Mus musculus DHC
Homo sapiens DHC
Consensus

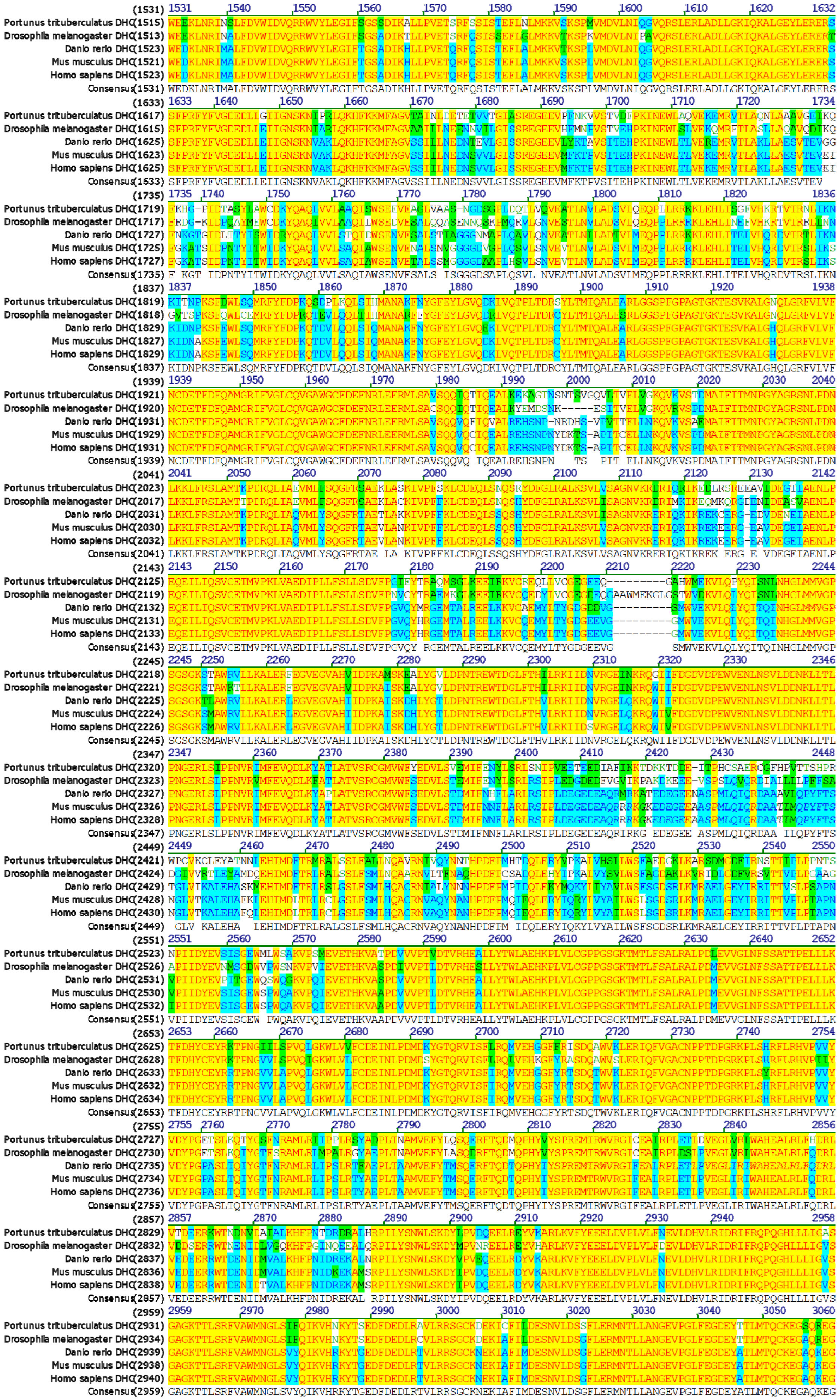
Portunus trituberculatus DHC
Drosophila melanogaster DHC
Danio rerio DHC
Mus musculus DHC
Homo sapiens DHC
Consensus
1531
3060

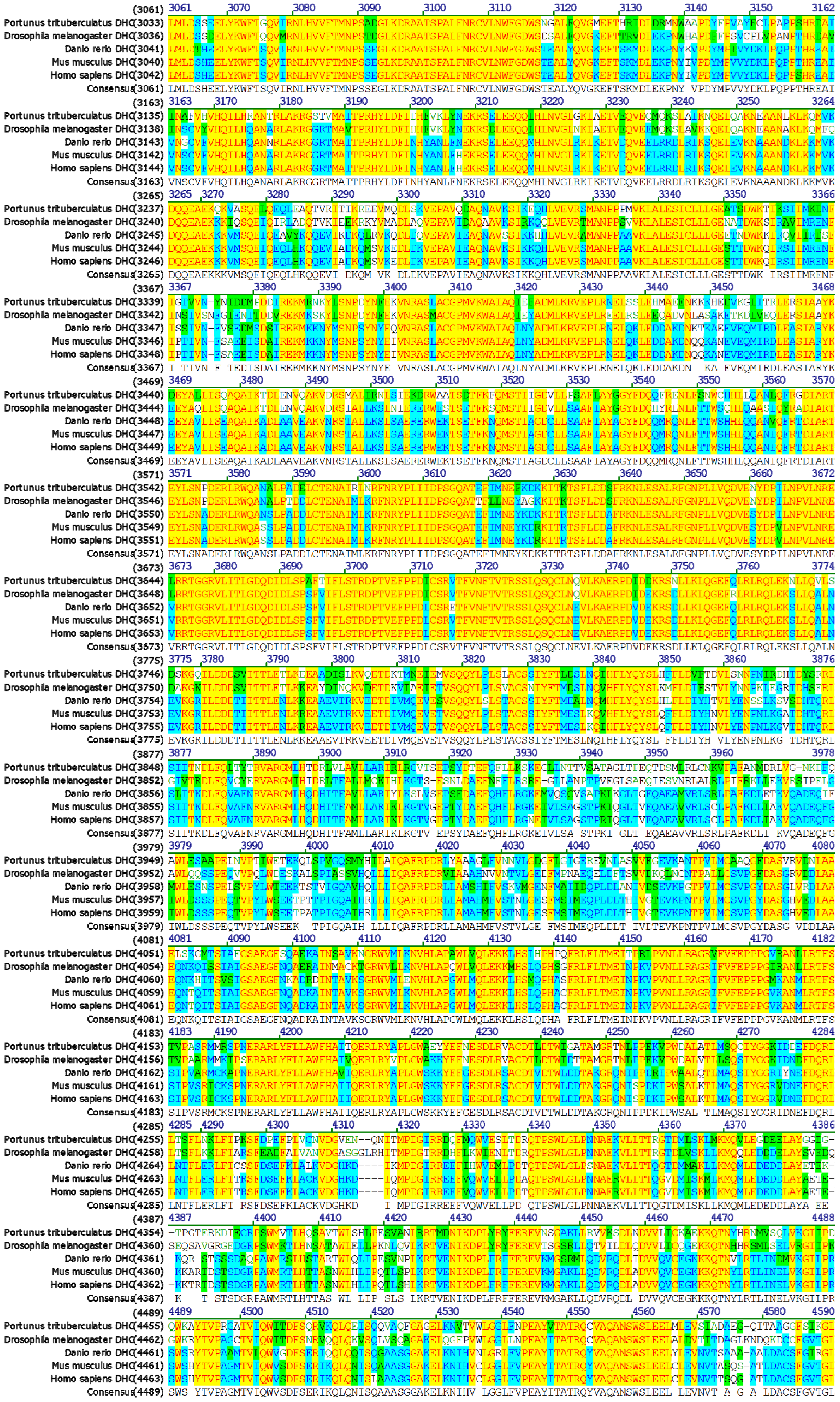

Portunus trituberculatus DHC
Drosophila melanogaster DHC
Danio rerio DHC
Mus musculus DHC
Homo sapiens DHC
Consensus

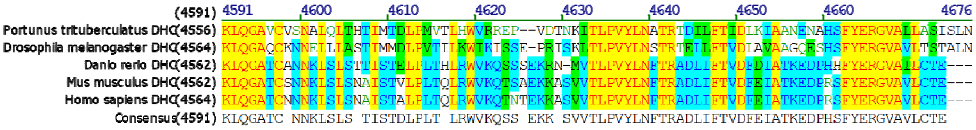

Figure S2： Multiple sequence alignment of protein sequences of *Pt-dhc*.
